# Patients with coronary artery disease after acute myocardial infarction: effects of continuous enrollment in a structured Disease Management Program on adherence to guideline-recommended medication, health care expenditures, and survival

**DOI:** 10.1007/s10198-020-01158-z

**Published:** 2020-02-01

**Authors:** Florian Kirsch, Christian Becker, Anja Schramm, Werner Maier, Reiner Leidl

**Affiliations:** 1 Institute of Health Economics and Health Care Management, Helmholtz Zentrum München, Neuherberg, Germany; 2 Munich School of Management and Munich Center of Health Sciences, Ludwig-Maximilians-Universität, Munich, Germany; 3 AOK Bayern, Service Center of Health Care Management, Regensburg, Germany

**Keywords:** AMI, DMP CAD, Health care expenditures, Survival, Guideline-based medication, I11, I18, H51

## Abstract

**Objective:**

Acute myocardial infarction (AMI) carries increased risk of mortality and excess costs. Disease Management Programs (DMPs) providing guideline-recommended care for chronic diseases seem an intuitively appealing way to enhance health outcomes for patients with chronic conditions such as AMI. The aim of the study is to compare adherence to guideline-recommended medication, health care expenditures and survival of patients enrolled and not enrolled in the German DMP for coronary artery disease (CAD) after an AMI from the perspective of a third-party payer over a follow-up period of 3 years.

**Methods:**

The study is based on routinely collected data from a regional statutory health insurance fund (*n* = 15,360). A propensity score matching with caliper method was conducted. Afterwards guideline-recommended medication, health care expenditures, and survival between patients enrolled and not enrolled in the DMP were compared with generalized linear and Cox proportional hazard models.

**Results:**

The propensity score matching resulted in 3870 pairs of AMI patients previously and continuously enrolled and not enrolled in the DMP. In the 3-year follow-up period the proportion of days covered rates for ACE-inhibitors (60.95% vs. 58.92%), anti-platelet agents (74.20% vs. 70.66%), statins (54.18% vs. 52.13%), and β-blockers (61.95% vs. 52.64%) were higher in the DMP group. Besides that, DMP participants induced lower health care expenditures per day (€58.24 vs. €72.72) and had a significantly lower risk of death (HR: 0.757).

**Conclusion:**

Previous and continuous enrollment in the DMP CAD for patients after AMI is a promising strategy as it enhances guideline-recommended medication, reduces health care expenditures and the risk of death.

**Electronic supplementary material:**

The online version of this article (10.1007/s10198-020-01158-z) contains supplementary material, which is available to authorized users.

## Introduction

Although mortality has decreased steadily in recent decades and survival rates of patients have increased [1], cardiovascular disease (CVD) remains one of the leading causes of mortality and morbidity in industrialized countries [2]. Acute myocardial infarction (AMI), a common manifestation of CVD in the elderly, carries increased risk of mortality, morbidity, and excess costs [3–7]. Disease management, which is increasingly being implemented in health care systems all over the world [8–10], seems an intuitively appealing way to improve quality, reduce the cost of care, and to enhance health outcomes for patients with chronic conditions such as AMI [8, 11]. Quality of care is expected to improve within DMPs through the implementation of evidence-based clinical practice. For example, by means of guideline-oriented health care provision, care protocols, and formulary lists of effective drugs, but also by improving coordination among different providers, and by assuring integration and comprehensiveness of care [12]. In Germany, the first two DMPs (type 2 diabetes and breast cancer) were implemented nationwide in 2002, the DMP for coronary artery disease (CAD) followed as third on May 1, 2003 [12]. Today around 1.8 million people with chronic illness are enrolled in the DMP CAD [13]. Contrary to other countries, in which DMPs were implemented with a focus on scientific evidence, in Germany, DMP rollout was accompanied by an elaborate legal framework that involved quality-of-care requirements, a strict accreditation process, and strong financial incentives for statutory health insurance funds to set up programs [14]. Five studies [15–19] evaluated CAD DMPs in Germany all using survey data. These studies assessed influence of patient characteristics [15], educational attainment and area deprivation [16], or the horizontal inequity indices and socio-economic status [17] on DMP enrollment. Furthermore, the influence of DMP enrollment on survival and guideline care [18] or quality of health care services and quality of health outcomes [19] were measured. To our knowledge, this is the first study evaluating the impact of enrollment in the DMP CAD using German statutory health insurance fund’s data. The aim of the study is to compare guideline-recommended medication, health care expenditures, and survival of patients previously and continuously enrolled and not enrolled in the German DMP for coronary artery disease (CAD) after an AMI, from the perspective of a third-party payer over a follow-up period of 3 years.

## Methods

### Data

The analysis is based on pseudonymized claims data provided by the Allgemeine Ortskrankenkasse Bayern (AOK Bayern), a large regional statutory health insurance fund in the German federal state of Bavaria, covering the years 2008–2014. Confirmed by the ethics committee of the State Chamber of Physicians of Bavaria, no ethical approval was required for this study.

### Study population

Individuals were included in the study if they had a hospitalization with a main discharge diagnosis of AMI (ICD-10 I21) between January 1, 2009 and December 31, 2011. AMIs before 2009 were excluded because hierarchical morbidity group (HMG) compensations, used as a control variable for morbidity, were not available. The HMG groups and their compensations relate to 80 costly chronic diseases and serious illnesses for which, the average health care expenditure per insured person exceeds the average health care expenditure of all insured persons by at least 50%. The HMG compensation are the ceteris paribus 1-year follow-up costs of the HMG group from a regression analysis [20]. In total, the HMG compensations can be seen as a differentiated comorbidity index. In our analysis we used the HMG groups in the year before index date, that the compensations reflect the predicted costs in the first year after the index date. AMIs after 2011 were excluded to guarantee a 3-year follow-up period. Patients were excluded if they died within 30 days after AMI, to avoid a negative overestimation of proportion of days covered (PDC) rates. As further inclusion criterion, patients had to be insured with the AOK Bayern continuously for at least 1 year before and 3 years after hospitalization, unless they died. Finally, patients were excluded as candidates for the control group if they were enrolled in the DMP CAD during the year before AMI or during the 3-year follow-up period.

### Outcome measures

Primary outcome measures were adherence rates based on PDCs, the average overall costs in euros (€) per person per day insured, and survival in days.

Adherence to guideline-based secondary prevention after AMI recommended in the German National Disease Management (NVL) for CAD [21] was assessed through the anatomical therapeutic chemical (ATC) classification system for: anti-platelet agents (B01A), statins (C10), β-blockers (C07), and angiotensin converting enzyme (ACE) inhibitors (C09A and C09B). Adherence rates were calculated using the PDC in the observation period, based on the total number of days supplied for filled prescriptions, given the number of defined daily doses (DDDs) per prescription. DDDs were supplied by the scientific institute of the AOK (‘WIdO’) based on a German adaption of the WHO database. If there were any discrepancies between the ‘WIdO’ DDDs and the DDD recommendations of the national guidelines [21], then the dosage from the national guidelines was used. In case of hospitalizations, it was assumed that drugs were supplied by the hospital and thus the number of days that needed to be covered was reduced by the length of hospital stays.

The health care expenditures analysis was based on routine data on individual level expenditure for filed claims for hospital, outpatient care, medication, rehabilitation (if covered by AOK Bayern), and remedies. Costs were calculated by summing up every patient’s costs by category and year and dividing them by the number of days the patient was insured in that period. A time-related outcome measure was selected in order to exclude bias from potential differences in mortality between patients enrolled and not enrolled in the DMP CAD. All costs were inflated to the year 2014, using the inflation rate as reported for Germany by the Organisation for Economic Co-operation and Development (OECD).

In order to calculate survival rates, days after myocardial infarction until death or end of the 3-year follow-up period were counted.

### Statistical analysis

Propensity score matching was conducted to elicit a control group of DMP participants who matched characteristics of DMP CAD participants with regard to the class variables “sex”, “smoking status”, “obesity”, “angina pectoris”, “arterial occlusive disease”, “dyslipidemia”, “congestive heart failure”, “arterial hypertonia”, “New York Heart Association (NYHA) state”, enrollment in “DMP COPD”, and “DMP type 2 diabetes”, “stent in the year before AMI”. Further, the continuous variables “age”, “German Index of Multiple Deprivation 2010 (GIMD 2010)” on district level [22], “HMG compensations per day”, and “length of index hospitalization” at baseline. A 1:1 matching was conducted on the logit of the propensity score using calipers of width equal to 0.2 of the standard deviation of the logit of the propensity score [23]. The “caliper” matching tends to result in estimates of treatment effect with less bias compared to other methods and has among the best performance when assessed using mean squared error [24].

Standardized mean differences were used for descriptive statistics to illustrate population characteristics for patients enrolled and not enrolled in the DMP CAD.

Generalized linear models (GLM) with beta distribution, for adherence rates (PDC rates were measured as percentage between 0 and 1) and gamma distribution for health care expenditures, and log-link were used to estimate the influence of DMP CAD enrollment on PDC rates and health care expenditures in euros per person per day [25, 26]. Models included the same covariates as in the propensity score matching. Separate cost analyses were conducted for each category of health care expenditures and year. Confidence intervals and *p* values for PDC rates and cost differences were derived by bootstrapping the original data set using 1000 replications [27].

Survival analyses were performed using Cox proportional hazards regressions. The first Cox proportional hazard regression model considered the same covariates as the cost analysis and the analysis of PDC rates. Further, as known from the literature that adherence to guideline-recommended medication has a protective effect on death in patients after AMI [18], an extended model was estimated that included the additional covariates of PDC rates for anti-platelet agents, statins, β-blockers, and ACE-inhibitors. To evaluate whether the proportional hazards assumption was met, Kaplan–Meier estimates of the survival functions were checked for parallelism of patients enrolled and not enrolled in the DMP. Furthermore, for all covariates, correlation of Schoenfeld residuals with survived days was examined, and the Kolmogorov–Smirnov supreme test [28] was conducted. In a sensitivity analysis, the covariates violating the proportional hazard assumption were considered, as they were additionally included in the basic and extended Cox proportional hazard model with time dependency [29–31]. A stratification for covariates violating the proportional hazard assumption did not seem meaningful, as a stratum with only one observation could not be excluded [29]. In a further sensitivity analysis we calculated the Charlson index and added it to both groups after matching and re-run the analysis by changing the variable HMG compensations against Charlson index for the base case analysis.

All analyses were performed using the SAS statistical package version 9.4 (SAS Institute, Cary, NC, USA).

## Results

### Study population

**Fig. 1:**
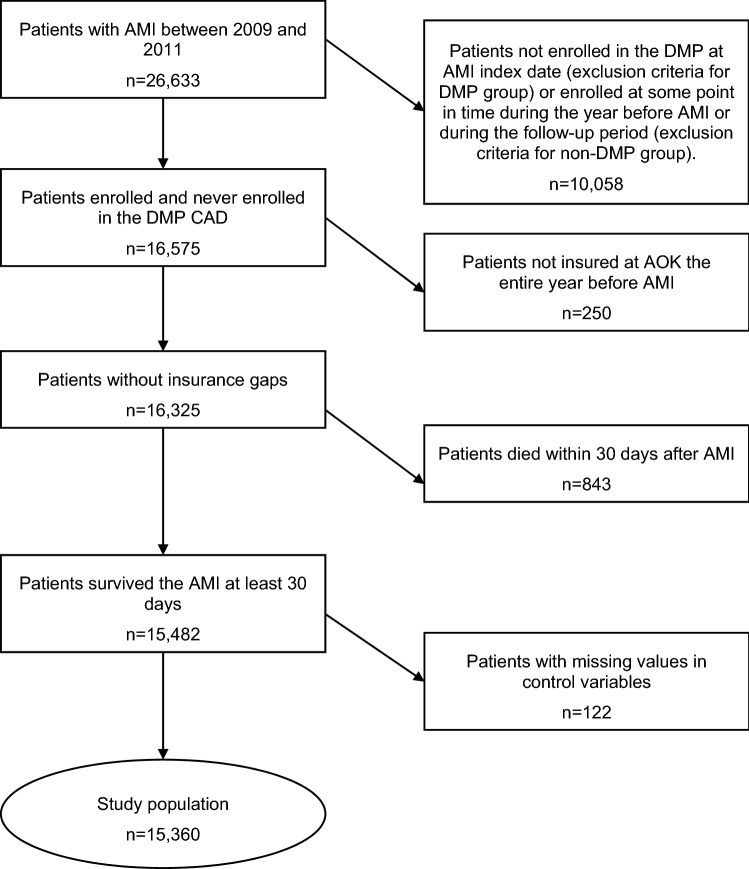
Patient selection

The selection of the study population is described in Fig. 1. The data set consisted of 26,633 patients who were discharged from hospital with a diagnosis of AMI between 2009 and 2011. Of these, 10,058 patients were excluded as they were not enrolled in the DMP at the AMI index hospitalization, but enrolled at some point during the year before AMI or during the 3-year follow-up period. Further, 250 patients were not insured the entire year before AMI, in addition, 843 persons died within the first 30 days after index AMI. Finally, 122 patients were excluded as they had missing values in covariates. Therefore, the study population consisted of 15,360 patients, including 4100 patients enrolled and 11,260 patients not enrolled in the DMP.

### Propensity score matching

The propensity score matching yielded 3870 pairs of patients enrolled and not enrolled in the DMP CAD. Baseline characteristics before and after matching are presented in Table 1. For balance assessment, the standardized mean differences before and after matching were measured. After matching, only the standardized mean difference of HMG compensations (0.161) were above the threshold of 0.10 [32, 33].

**Table 1:** Balance assessment propensity score matching

Variables	Before matching			After matching		
	No DMP	DMP	SMD	No DMP	DMP	SMD
	*n* = 11,260	*n* = 4100		*n* = 3870	*n* = 3870	
Sex						
Female	**5188 (46.07%)**	**1359 (33.15%)**	**− 0.267**	1323 (34.19%)	1317 (34.03%)	**− **0.003
Smokers	528 (4.69%)	283 (6.90%)	0.095	219 (5.66%)	266 (6.87%)	0.050
Obesity	**1466 (13.02%)**	**719(17.54%)**	**0.126**	618 (15.97%)	653 (16.87%)	0.024
Angina pectoris	**482 (4.28%)**	**493 (12.02%)**	**0.286**	358 (9.25%)	399 (10.31%)	0.036
Arterial occlusive disease	**1053 (9.35%)**	**1139 (27.78%)**	**0.488**	884 (22.84%)	934 (24.13%)	0.030
Dyslipidemia	**1152 (10.23%)**	**882 (21.51%)**	**0.312**	689 (17.80%)	724 (18.71%)	0.023
Congestive heart failure	**1359 (12.07%)**	**679 (16.56%)**	**0.129**	582 (15.04%)	605 (15.63%)	0.016
Arterial hypertonia	**2803 (24.89%)**	**1380 (33.66%)**	**0.194**	1176 (30.39%)	1221 (31.55%)	0.025
NYHA						
0	7401 (65.73%)	2670 (65.12%)	0.079	2513 (64.94%)	2529 (65.35%)	0.090
1	149 (1.32%)	65 (1.59%)		50 (1.29%)	61 (1.58%)	
2	550 (4.88%)	238 (5.80%)		189 (4.88%)	223 (5.76%)	
3	1225 (10.88%)	504 (12.29%)		422 (10.90%)	469 (12.12%)	
4	1935 (17.18%)	623 (15.20%)		696 (17.98%)	588 (15.19%)	
DMP COPD	**265 (2.35%)**	**278 (6.78%)**	**0.213**	196 (5.06%)	209 (5.40%)	0.015
DMP type 2 diabetes	**2452 (21.78%)**	**1711 (41.73%)**	**0.439**	1532 (39.59%)	1522 (39.33%)	**− **0.005
Stent before	**1177 (10.45%)**	**913 (22.27%)**	**0.324**	743 (19.20%)	775 (20.03%)	0.021
Length index hospitalization	11.50 [11.33;14.67]	10.93 [10.68;11.18]	**− **0.051	10.85 [10.59;11.10]	11.00 [10.74;11.26]	0.012
Age	73.14 [72.91;73.40]	73.66 [73.35;73.97]	**− **0.037	74.35 [73.99;74.71]	73.65 [73.32;73.97]	**− **0.098
HMG compensations	**€11.59 [€11.22;€11.97]**	**€14.24 [€13.68;€14.80]**	**0.421**	**€14.83 [€14.06;€15.61]**	**€13.90 [€13.33;€14.46]**	**0.161**
GIMD 2010	14.32 [14.18;14.46]	14.62 [14.39;14.85]	0.058	14.70 [14.45;15.60]	14.61 [14.38;14.85]	0.006

Bold values indicate SMD > 0.10

*NYHA* New York Heart Association, *DMP* Disease Management Program, *CAD* coronary artery disease, *COPD* chronic obstructive disease, *HMG* Hierarchical Morbidity Group, *GIMD 2010* German Index of Multiple Deprivation 2010,* SMD* standardized mean difference

### Guideline-recommended medication

PDC rates for anti-platelet agents (76.43% vs. 70.66%), statins (54.18% vs. 52.13%), and ACE-inhibitors (60.95% vs. 58.92%) over the 3-year follow-up period and for β-blockers (61.95% vs. 52.64%) over the first year after AMI were all higher in the DMP group, only the difference for β-blockers was statistically significant (see also Supplement Table 2).

### Health care expenditures

Table 2 shows the adjusted mean health care expenditures per day per person for the three observation years. Health care expenditures per person per day of €72.72 were incurred in non-DMP group and €58.24 in the DMP group (*p* < 0.001). Hospitalization costs far exceeded costs of medication, outpatient care, rehabilitation, and remedies for each observation year. The cost difference between the DMP and non-DMP group appeared to be mainly driven by higher inpatient expenditures in the first year after AMI. After differentiating by year, the cost differences between patient groups appeared to converge over time. Compared to the first year after AMI, in which significantly higher health care expenditures arise in the non-DMP group with regard to hospitalization (*p* < 0.001), rehabilitation (*p* < 0.05) and remedies (*p* < 0.001), in year two only costs for remedies (*p* < 0.001) and in year three only costs for ambulatory care (*p* < 0.05) were significantly different.

**Table 2:** Health care expenditures according to DMP enr ollment

	Non-DMP		DMP	
Health care expenditures all 3 years***	***N* = 3870**	**€72.72 [€67.56;€78.38]**	***N* = 3870**	**€58.24 [€53.36;€63.81]**
Health care expenditures year 1***	***N* = 3870**	**€85.45 [€80.28;€90.84]**	***N* = 3870**	**€71.18 [€66.55;€76.26]**
Health care expenditures year 2	*N* = 3106	€22.98 [€20.69;€25.34]	*N* = 3337	€24.05 [€21.79;€26.45€]
Health care expenditures year 3	*N* = 2684	€19.45 [€17.42;€21.73]	*N* = 3008	€22.04 [€19.98;€24.53€]
Outpatient care all 3 years	*N* = 3870	€2.75 [€2.20;€3.48€]	*N* = 3870	€3.06 [€2.38;€3.90€]
Outpatient care year 1	*N* = 3870	€2.75 [€2.14;€3.57]	*N* = 3870	€2.95 [€2.20;€3.90]
Outpatient care year 2	*N* = 3106	€2.37 [€1.75;€3.34]	*N* = 3337	€2.65 [€1.94;€3.83]
Outpatient care year 3*	*N* = 2684	€2.14 [€1.50;€3.19]	*N* = 3008	€3.16 [€2.17;€4.89]
Medication all 3 years	*N* = 3870	€4.17 [€3.92;€4.45]	*N* = 3870	€3.95 [€3.68;€4.23]
Medication year 1	*N* = 3870	€4.80 [€4.49;€5.15]	*N* = 3870	€4.63 [€4.33;€4.92]
Medication year 2	*N* = 3106	€3.18 [€2.93;€3.47]	*N* = 3337	€3.21 [€2.90;€3.56]
Medication year 3	*N* = 2684	€2.71 [€2.44;€3.03]	*N* = 3008	€2.66 [€2.42;€3.01]
Hospitalization all 3 years***	***N* = 3870**	**€61.85 [€56.73;€67.43]**	***N* = 3870**	**€48.60 [€44.00;€54.07]**
Hospitalization year 1***	***N* = 3870**	**€72.86 [€67.87;€77.97]**	***N* = 3870**	**€59.92 [€55.32;€64.77]**
Hospitalization year 2	*N* = 3106	€16.03 [€14.27;€17.85]	*N* = 3337	€16.46 [€14.65;€18.44]
Hospitalization year 3	*N* = 2684	€13.55 [€11.85;€15.28]	*N* = 3008	€14.99 [€13.37;€16.83]
Rehabilitation all 3 years***	***N* = 3870**	**€2.39 [€2.16;€2.65]**	***N* = 3870**	**€1.91 [€1.72;€2.11]**
Rehabilitation year 1*	***N* = 3870**	**€3.52 [€3.27;€3.77]**	***N* = 3870**	**€3.14 [€2.93;€3.37]**
Rehabilitation year 2	*N* = 3106	€0.35 [€0.28;€0.43]	*N* = 3337	€0.45 [€0.36;€0.56]
Rehabilitation year 3	*N* = 2684	€0.30 [€0.24;€0.37]	*N* = 3008	€0.35 [€0.27;€0.43]
Remedies all 3 years***	***N* = 3870**	**€1.46 [€1.34;€1.61]**	***N* = 3870**	**€1.11 [€1.01;€1.22]**
Remedies year 1***	***N* = 3870**	**€1.49 [€1.35;€1.64]**	***N* = 3870**	**€1.11 [€1.01;€1.22]**
Remedies year 2***	***N* = 3106**	**€0.90 [€0.80;€1.02]**	***N* = 3337**	**€0.70 [€0.62;€0.78]**
Remedies year 3	*N* = 2684	€0.80 [€0.70;€0.96]	*N* = 3008	€0.81 [€0.70;€0.95]

*p < 0.05; **p < 0.01; ***p < 0.001

Confidence intervals and *p* values of the cost differences were derived by bootstrapping the original data set with 1000 replications [26]

### Survival

A Cox proportional hazard model was used for the survival analysis. Testing the proportional hazard assumption (Supplement Fig. 1 and Tables 2 to 5), the curves of the Kaplan–Meier estimates were parallel (Supplement Fig. 1). The correlation analysis of the Schoenfeld residuals for all covariates with time indicated a problem with length of index hospitalization (*p* = 0.0199) and HMG compensations (*p* = 0.0107) in the basic model (Supplement Table 2), and with length of index hospitalization (*p* = 0.0301), HMG compensations (*p* = 0.0030), PDC rates of ACE-inhibitors (*p* < 0.0001), and β-blockers (*p* < 0.0001) in the extended model (Supplement Table 4). The Kolmogorov–Smirnov supreme test yielded similar results regarding length of index hospitalization (*p* = 0.0180) and HMG compensations (*p* = 0.0010) in the basic model (Supplement Table 3) and, in the extended model (Supplement Table 5), for length of index hospitalization (*p* = 0.0280), HMG compensation (*p* < 0.0001), PDC rates of ACE-inhibitors (*p* < 0.0001), β-blockers (*p* < 0.001), and anti-platelet agents (*p* = 0.0280).

**Fig. 2:**
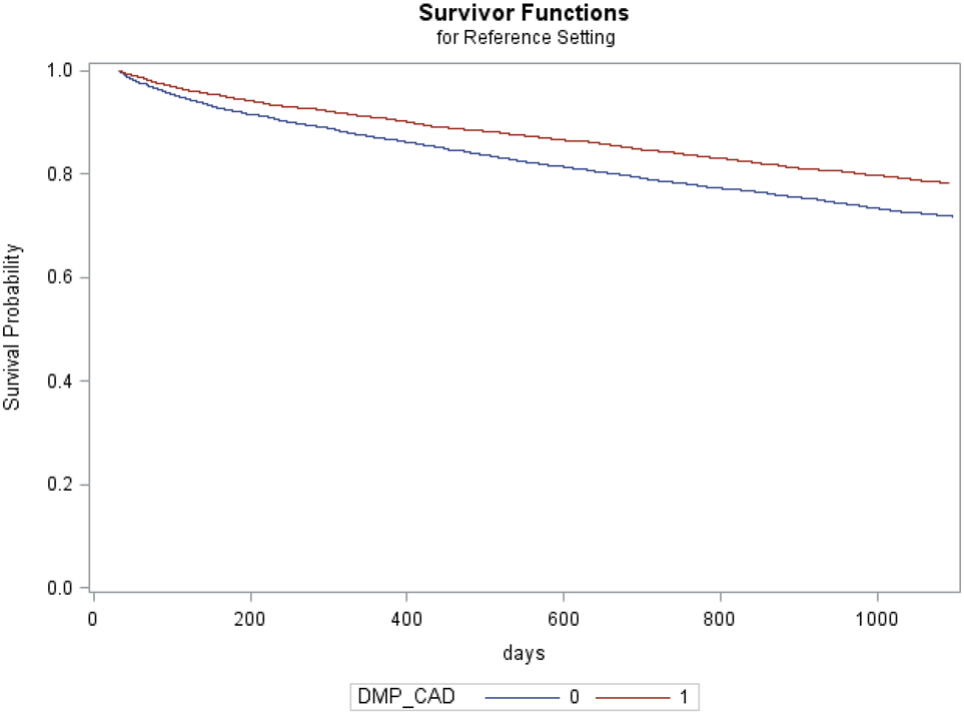
Adjusted survivor function of DMP CAD versus non-DMP CAD

In the base case survival analysis (Table 3 and Fig. 2) enrollment in the DMP CAD was associated with highly decreased risk of death as it had a hazard rate (HR) of 0.757 (*p* < 0.001) compared to the pairs in the non-DMP group. Additional enrollment in the DMP COPD HR = 1.319 (*p* < 0.001) and DMP type 2 diabetes HR = 1.124 (*p* < 0.01) increased risk of death. Moreover, age HR = 1.064 (*p* < 0.001), HMG compensation HR = 1.010 (*p* < 0.001), NYHA states 3 HR = 1.435 (*p* < 0.001), NYHA 4 HR = 1.754 (*p* < 0.001), congestive heart failure HR = 1.409 (*p* < 0.001), arterial occlusive disease HR = 1.208 (*p* = 0.0014), arterial hypertonia HR = 1.124 (*p* < 0.001), length of index hospitalization HR = 1.020 (*p* < 0.001), and smoking HR = 1.698 (*p* < 0.001) were associated with increased risk of death. Angina pectoris HR = 0.810 (*p* = 0.0042) and being female HR = 0.832 (*p* < 0.001) appeared to be protective.

**Table 3:** Basic proportional hazard model

Parameters	DF	Estimate	StdErr	ChiSq	ProbChiSq	Hazard ratio (CI 95%)
Sex	1	− **0.18377**	**0.04261**	**18.5999**	**<0.0001**	**0.832 (0.765–0.905)**
Smokers	1	**0.52947**	**0.08533**	**38.5042**	**<0.0001**	**1.698 (1.437–2.007)**
Obesity	1	− 0.02643	0.05499	0.2310	0.6308	0.974 (0.874–1.085)
Angina pectoris	1	**− 0.21085**	**0.07364**	**8.1975**	**0.0042**	**0.810 (0.701–0.936)**
Arterial occlusive disease	1	**0.18919**	**0.05909**	**10.2516**	**0.0014**	**1.208 (1.076–1.357)**
Dyslipidemia	1	− 0.03745	0.05850	0.4099	0.5220	0.963 (0.859–1.080)
Congestive heart failure	1	**0.34277**	**0.05477**	**39.1721**	**<0.0001**	**1.409 (1.265–1.568)**
Arterial hypertonia	1	**0.11662**	**0.05179**	**5.0712**	**0.0243**	**1.124 (1.015–1.244)**
NYHA 1	1	0.03473	0.16943	0.0420	0.8376	1.035 (0.743–1.443)
NYHA 2	1	− 0.01271	0.09536	0.0178	0.8940	0.987 (0.819–1.190)
NYHA 3	1	**0.36134**	**0.05859**	**38.0289**	**<0.0001**	**1.435 (1.280–1.610)**
NYHA 4	1	**0.56168**	**0.04955**	**128.4801**	**<0.0001**	**1.754 (1.591–1.932)**
DMP CAD	1	**− 0.27874**	**0.04005**	**48.4325**	**<0.0001**	**0.757 (0.700–0.819)**
DMP COPD	1	**0.27655**	**0.08045**	**11.8165**	**0.0006**	**1.319 (1.126–1.544)**
DMP type 2 diabetes	1	**0.11648**	**0.04077**	**8.1633**	**0.0043**	**1.124 (1.037–1.217)**
Stent before (binary)	1	0.08420	0.05261	2.5613	0.1095	1.088 (0.981–1.206)
Length index hospitalization	1	**0.01936**	**0.00209**	**86.0518**	**<0.0001**	**1.020 (1.015–1.024)**
Age	1	**0.06242**	**0.00259**	**580.1605**	**<0.0001**	**1.064 (1.059–1.070)**
HMG compensations	1	**0.01042**	**0.00066**	**249.4045**	**<0.0001**	**1.010 (1.009–1.012)**
GIMD 2010	1	0.00049	0.00261	0.0351	0.8514	1.000 (0.995–1.006)

Bold values indicate* p* < 0.05

*NYHA* New York Heart Association, *DMP* Disease Management Program, *CAD* coronary artery disease, *COPD* chronic obstructive disease, *HMG* Hierarchical Morbidity Group, *GIMD 2010* German Index of Multiple Deprivation 2010

Including the percentage rate of PDCs for each of the four guideline-recommended medications in an extended Cox proportional hazard model yielded quite similar results (Table 4). The hazard rates of the covariates included in the basic model only changed slightly in both directions. For the newly included covariates, only a higher PDC rate of statins seemed to reduce the HR (0.098; *p* = 0.0011), while a higher PDC rate for ACE-inhibitors (HR = 1.003; *p* = <0.0001) and anti-platelet-agents (HR = 1.005; *p* = 0.0001) appeared to increase the risk, and β-blockers (*p* = 0.1205) did not have a significant influence on death.

**Table 4:** Proportional hazard model with guideline-recommended medication

Parameters	DF	Estimate	StdErr	ChiSq	ProbChiSq	Hazard ratio (CI 95%)
Sex	1	**− 0.17748**	**0.04277**	**17.2203**	**<0.0001**	**0.837 (0.770–0.911)**
Smokers	1	**0.52461**	**0.08553**	**37.6245**	**<0.0001**	**1.690 (0.429–1.998)**
Obesity	1	− 0.05155	0.05503	0.8776	0.3489	0.950 (0.853–1.058)
Angina pectoris	1	**− 0.21522**	**0.07378**	**8.5099**	**0.0035**	**0.806 (0.698–0.932)**
Arterial occlusive disease	1	**0.17618**	**0.05939**	**8.8004**	**0.0030**	**1.193 (1.062–1.340)**
Dyslipidemia	1	− 0.02435	0.05886	0.1712	0.6790	0.976 (0.870–1.095)
Congestive heart failure	1	**0.33437**	**0.05491**	**37.0829**	**<0.0001**	**1.397 (1.255–1.556)**
Arterial hypertonia	1	**0.10932**	**0.05202**	**4.4171**	**0.0356**	**1.116 (1.007–1.235)**
NYHA 1	1	0.07305	0.16939	0.1860	0.6663	1.076 (0.772–1.499)
NYHA 2	1	− 0.00172	0.09540	0.0003	0.9856	0.998 (0.828–1.204)
NYHA 3	1	**0.36360**	**0.05864**	**38.4525**	**<0.0001**	**1.439 (1.282–1.614)**
NYHA 4	1	**0.54481**	**0.04962**	**120.5602**	**<0.0001**	**1.724 (1.564–1.900)**
DMP CAD	1	**−0.27154**	**0.04024**	**45.5422**	**<0.0001**	**0.762 (0.704–0.825)**
DMPCOPD	1	**0.27996**	**0.08072**	**12.0295**	**0.0005**	**1.323 (1.129–1.550)**
DMP type 2 diabetes	1	**0.09653**	**0.04094**	**5.5604**	**0.0184**	**1.101 (1.016–1.193)**
Stent before (binary)	1	**0.10501**	**0.05309**	**3.9131**	**0.0479**	**1.111 (1.001–1.233)**
Length index hospitalization	1	**0.01946**	**0.00209**	**87.1523**	**<0.0001**	**1.020 (1.015–1.024)**
Age	1	**0.06235**	**0.00267**	**543.9483**	**<0.0001**	**1.064 (1.059–1.070)**
HMG compensations	1	**0.01012**	**0.00067**	**228.2524**	**<0.0001**	**1.010 (1.009–1.011)**
GIMD 2010	1	**−** 0.00083	0.00263	0.0998	0.7521	0.999 (0.994–1.004)
PDC ace-inhibitors	1	**0.00292**	**0.00057**	**26.5957**	**<0.0001**	**1.003 (1.002–1.004)**
PDC β-blockers	1	0.00096	0.00062	2.4112	0.1205	1.001 (1.000–1.002)
PDC statins	1	**−0.00201**	**0.00062**	**10.5721**	**0.0011**	**0.998 (0.997–0.999)**
PDC anti-platelet-agents	1	**0.00541**	**0.00054**	**101.0163**	**<0.0001**	**1.005 (1.004–1.006)**

Bold values indicate* p* < 0.05

*NYHA* New York Heart Association, *DMP* Disease Management Program, *CAD* coronary artery disease, *COPD* chronic obstructive disease, *HMG* Hierarchical Morbidity Group, *GIMD 2010* German Index of Multiple Deprivation 2010, *PDC* proportion of days covered

These results ignored the proportional hazards assumption violation of length of index hospitalization and HMG compensations in the basic model, and in the extended model. To overcome this shortage, further models were the covariates violating the proportional hazard assumption were multiplied with time and incorporated into the model.

For the basic model (Supplement Table 6), the significance level of all variables stayed the same and the HR only changed minimal in both directions. The new incorporated variables were both significant (days*length of index hospitalization *p* < 0.0001; days*HMG compensation *p* = 0.0065) but have a HR of 1.000.

In the extended model (Supplement Table 7), the significance level of β-blockers changed from non-significant (*p* = 0.1205) to highly significant (*p* = 0.0002) with an HR of 1.004. All newly included covariates besides the time-dependent variable of anti-platelet-agents became statistically significant and had a HR of 1.000. The significance level of the already included covariates did not change and the HR only changed slightly in both directions.

In a sensitivity analysis, we included the Charlson comorbidity index instead of the HMG compensations as a covariate in the base case Cox proportional hazard model. The Charlson index with a mean of 5.27 [5.18–5.36] in the non-DMP and 5.00 [4.91–5.09] was well balanced with a standardized mean difference of − 0.082 (even though it was not considered in the propensity score matching). The model estimates remain quite similar. Only the significance value from arterial hypertonia changed from originally significant to non-significant. The hazard rates for the covariates only changed slightly and the hazard rate for the Charlson index was 1.147 (1.130–1.164) while the HMG compensation was 1.010 (1.009–1.012) (Supplement Table 8).

## Discussion

### Main results

Recently, claims data of statutory health insurance funds were used more often in analyses of health care provision. So far, this is still the first study evaluating the differences in adherence to guideline-recommended medications, health care expenditures, and survival of AMI patients enrolled and not enrolled in the DMP CAD using data of a statutory health insurance fund in Germany. All in all, propensity score matching seems a useful approach to cope with biased patient preselection [34] in observational studies. Also in our study differences in patient characteristics almost disappeared completely after matching. So we were able to show that being enrolled in the DMP CAD after AMI is a promising strategy as it is associated with enhanced guideline-recommended medication, lower total health care expenditures and reduced risk of death.

### Comparison of findings with literature

Medication usage in the DMP CAD was measured by Gapp et al. [19] and Stark et al. [18], based on survey data. While Gapp et al. [19] considered all persons enrolled in the DMP CAD, Stark et al. [18] included, similar to our study, only patients with a previous AMI. Both studies [18, 19] found a significantly higher usage rate of anti-platelet agents and statins in the DMP group. Additionally, Stark et al. [18] measured usage of ACE-inhibitors and percentage of people receiving guideline care (advice regarding diet, exercise or smoking within the last year and intake of β-blockers, statins, and agents acting on the renin–angiotensin system), which were both significantly higher in the DMP group. In our study, we also considered medication recommended for secondary prevention after AMI and found higher PDC rates regarding anti-platelet agents, statins, β-blockers, and ACE-inhibitors in the DMP group. Notably, in our analysis β-blockers were the only medication with a significant difference, but the only medication that was not statistically different in the study by Stark et al. [18]. One explanation might be that β-blockers are only recommended by medical guidelines in the first or rather in the first 2 years after AMI and in the study conducted by Stark et al. [18] the last AMI was 7.7 years ago in the DMP group and 9.6 years in the usual care group. When interpreting the results it should be considered that the publication from Gapp et al. [19] was in 2008 and Stark et al. [18] in 2014. Since then a higher percentage of patients is enrolled in the DMP CAD, which might have changed the patient characteristics in the DMP over time.

Whereas so far no economic analyses were conducted on health care expenditures for the DMP CAD in Germany, there are previous studies that focused on the DMPs COPD [35] and type 2 diabetes [13, 36–40]. In COPD, Achelrod et al. [35] conducted a study using data of a statutory health insurance fund and found that expenditures for hospitalization, ambulatory care and medication were significantly higher in the DMP group, leading to significantly higher total health care expenditures. Concerning type 2 diabetes, to date seven publications based on statutory health insurance claims data reported cost measures for the DMP type 2 diabetes [13, 36–40]. Five studies [13, 36, 38, 39] reported overall health care expenditures—all of these studies reported lower costs in the DMP group, but only in two studies [36, 38] the results were significant. Six studies [13, 36–40] reported expenditures for inpatient care, in all studies the hospitalization costs were lower in the DMP group, but significant in only four studies [36–39]. Ambulatory care was measured in two studies [38, 39] and in both studies ambulatory care costs were significantly higher in the DMP group. Regarding medications, four studies [13, 37, 39, 40] reported higher costs, two [39, 40] of them statistically significant, and two reported lower costs [36, 37]. Our results are seemingly in line with the results on health care expenditures in DMPs so far: we found also significantly lower overall health care expenditures in the DMP CAD group. In addition, we found significantly lower costs in the categories hospitalization, rehabilitation and remedies and lower costs for medication in the DMP group that was not significant. Only the cost of outpatient care was higher in the DMP group, but this result was not statistically significant.

Only Stark et al. [18] measured differences in mortality and found that DMP CAD participation reduced the mortality risk by 10% (HR = 0.90) (not statistically significant). Receiving guideline care showed instead a statistically significant reduction in all-cause mortality by almost 60% (HR = 0.41). We found a HR of 0.756 and 0.762, indicating that the risk of death is significantly reduced by DMP CAD enrollment. For guideline-recommended medication, we found mixed results: while only a higher PDC rate in statins reduced the risk of death, higher rates in ACE-inhibitors, antiplatelet-agents, and, in the sensitivity analyses of the extended model, also β-blockers, were significantly associated with a higher risk of death. One explanation could again be the difference in time since the considered patients’ last AMI. Another reason might be the difference in measurement of adherence: while we measured PDC rates which declined over the entire 3-year follow-up period, adherence in the study of Stark et al. [18] was based on patient-reported medication intake during the last week, which otherwise might cause a tendency for social desirable answers by patients. Furthermore, there might be selection bias in the study conducted by Stark et al. [18] as adherence was measured with a postal questionnaire, which the oldest and most severe ill patients were less likely to fill out and return. In our study, there might be reverse causation instead, implying that patients with greater severity of illness have higher PDC rates for the guideline-recommended medications.

As a surprising result, protective effects were discovered for angina pectoris and female gender in the survival analysis. For angina pectoris we identified several other studies which found a relationship estimating a prognostic value of preinfarction angina pectoris by indicating less extensive infarct size resulting in better short- and long-term survival [41–46]. The protective effect of being female on survival is less backed up by evidence. A German observational study found a higher unadjusted morality rate in hospital in women (10.8%) than in men (7.1%) [47]. We excluded patients who died in hospital, so these patients were not considered in the Cox proportional hazard model. Therefore, women after hospital discharge might have a better prognosis.

### Strengths and limitations

The study has some notable strengths. We conducted a propensity score matching with the caliper method, which eliminated many initial differences between the patients enrolled and not enrolled in the DMP. The propensity score matching worked well, because almost three times as many persons were available as potential controls than persons were enrolled. This elimination of structural differences was not possible in the survey-based publications on DMP CAD [18, 19].

Further, the data allowed us to consider a long period, 1 year before AMI and a 3-year follow-up period, which made it possible to include variables such as health care expenditures, HMG compensations or stent surgeries in the year before AMI. Additionally, medication stocks for the PDC rate calculation could be considered, resulting in a more realistic estimate of adherence.

Moreover, we included an area deprivation index on district level (German Index of Multiple Deprivation 2010) [48–50] as a proxy for individual socioeconomic status not sufficiently reflected in routine data of a German statutory health insurance fund. The approach entails the potential of misclassification bias, in that individuals are matched on the basis of an area-measure of socioeconomic status, which may differ from their individual status. However, studies evaluating the DMP CAD and compared individual socioeconomic factors between patients enrolled and not enrolled in the DMP and found only a significant positive impact of height of old-age pensions [15], but not of education [16–19] or income [15] on enrollment in the DMP. Nonetheless, it is difficult to estimate how this impacts the results, as the GIMD 2010 was well balanced after matching between the DMP and non-DMP group and was an insignificant factor in the Cox proportional hazard analyses.

Besides the strengths, some potential limitations of this study should be considered while interpreting the results.

Since patients are not randomized regarding either DMP participation or guideline-recommended medication, treatment choices may be based on selection bias [51]. The additional medical education requirements for physicians to offer DMP services might result in spillover effects of guideline-recommended treatment to patients not enrolled in the DMP [52], which cannot be ruled out by propensity score matching. Unfortunately, we were not able to quantify the spill-over effects because we did not know which patients were treated by which doctor, as we did not have information to identify the doctor’s office or the doctor itself.

The data underlying the covariates smoking and obesity were outpatient and inpatient ICD-10 codes for the DMP and non-DMP group, as information from the DMP documentation form were not available for the non-DMP group. According to the descriptive analysis, smoking might be prone to underreporting in ICD-10 codes. The prevalence for smoking was quite low at 5.66% and 6.87% in the non-DMP and DMP group, respectively. As we did not have height and weight from the DMP documentation form for all patients, we could not calculate the BMI. Therefore, we included a binary variable for obesity based on ICD-10 codes, which yet is less precise. The prevalence of obesity was 15.97% and 16.87% in the non-DMP and DMP group, respectively. Therefore, the effect of smoking and obesity might be underestimated in our statistical analysis.

Differences in severity of heart disease may impact results [53–58]. According to our data structure, we could not observe for how long persons suffer from CAD before AMI and we could not exclude the possibility that the index AMI was not the first AMI. We had data from January 1, 2008 to December 31, 2014 and we considered AMIs between January 1, 2009 and December 31, 2011. Therefore, we had an observation period before AMI from 365 days up to 1460 days, depends on index AMI date, for that period a prior AMI could be excluded. However, we can only estimate to a limited extent whether patients enrolled in the DMP may have a longer disease history with CAD and AMIs than patients not enrolled in the DMP. Nevertheless, we have tried to control for severity in general and heart related diseases in our statistical analyses. In the propensity score matching and statistical models we used HMG compensations, which reflect the general severity of comorbidity and is a quite accurate predictor of health care expenditures [20], and the heart related diseases angina pectoris, arterial occlusive disease, dyslipidemia, congestive heart failure, arterial hypertonia, and NYHA which might still not fully reflect the considered patients’ severity of heart disease and general state of health.

Pharmacy-dispensing data were used as a measure of PDC, which does not allow definite judgment as to whether patients had actually taken the medication collected at the pharmacy. However, pharmacy refill records have been argued to reflect a patient’s active decision to continue with therapy and the corresponding rates highly correlate with rates in electronic adherence monitoring [59].

Costs for rehabilitation were only included if the statutory health insurance was the third-party payer. Pension fund normally pays rehabilitations for persons below retirement age, and the employers’ liability pays for patients with occupational diseases. AMI is usually not an occupational disease and mean age of the study population was well above 70 years. Therefore, the bias resulting from this limitation should be small.

HMG compensations were used as a proxy for disease severity. Empirical results showed that, at an individual level, the HMG compensations achieved a predictive accuracy of about 24% in 2011 for health care expenditures [20]. The HMG compensations were designed to predict health care expenditures and not to predict survival. Therefore, HMG compensations may be a good covariate for cost estimates, but not as good for a survival analysis. Nevertheless, we used it for all analyses for consistency. Further, HMG compensations between DMP and non-DMP group were not balanced well after propensity score matching as the standardized mean difference of 0.161 was above the threshold of 0.10. It is hard to quantify how this difference in comorbidities influences the results. As the monthly HMG compensation is €0.93 higher in the non-DMP group the health care expenditures according to the diseases in the HMG compensation scheme should be €0.93 higher per month. However, leading to the conclusion that the morbidity is higher in the non-DMP group, which is also reflected in the difference in the Charlson index, might overestimate the protective influence of the enrollment in the DMP CAD on survival.

Clinical trials showed that the intake of anti-platelet agents [60–62], statins [63–65], ACE-inhibitors [66–68], and β-blockers [69–71] lower the risk of death after myocardial infarction, which could not be shown in our analyses as we had a mainly negative association of PDC rates and death. On one the hand, it is related to the steady decline of PDC rates in the years after AMI that patients who died earlier had higher PDC rates compared to patients who survived the complete follow-up period. On the other hand, this may be due to reverse causation, e.g., persons in a more severe general condition are more motivated to take their medication or see their physician more frequently and therefore receive more medication prescriptions. However, it seems that the persons included and the treatment by physicians in our study may differ from the strict patient selection and treatment plan of the study protocol in clinical trials, leading to different results. We do not expect that this reverse causation problem to influence the results in general, as the analysis was conducted after propensity score matching containing several covariates estimating disease severity; therefore, the DMP and non-DMP group should be affected in the same way.

## Conclusion

Results show that being enrolled in the DMP CAD appears to be a dominant strategy after AMI as it is associated with enhanced guideline-recommended medication, cost savings and prolonged life, while adherence to three out of four guideline-recommended medications after AMI was not associated with a lower mortality contrary to findings from randomized trials. Accordingly, a point, warranting further scrutiny in this context is why results based on real-world data of a statutory health insurance fund deliver diverse results from randomized clinical trials.

## Electronic supplementary material

Below is the link to the electronic supplementary material.

Supplementary material 1 (DOCX 60 kb)
