# Supplementary material for: Patients with coronary artery disease after acute myocardial infarction: effects of continuous enrollment in a structured Disease Management Program on adherence to guideline-recommended medication, health care expenditures, and survival
Source: Eur J Health Econ. 2020 Feb 1;21(4):607–19. doi: 10.1007/s10198-020-01158-z (PMC7214389; doi:10.1007/s10198-020-01158-z)
Supplement: Supplementary file 1 — Supplementary material 1 (DOCX 60 kb) [file 10198_2020_1158_MOESM1_ESM.docx]

**Supplement:**

**Supplement Fig. 1: Plot of ‘log-negative-log’ of the Kaplan-Meier estimator**


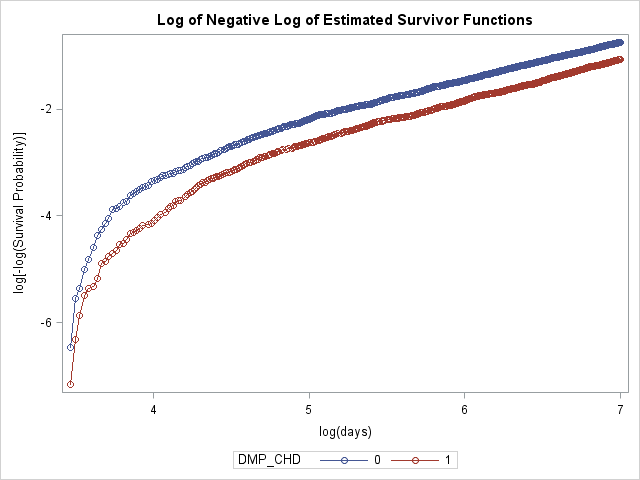


**Supplement Table 1: Differences in Adherence according to DMP enrollment**

| **PDC rates in percentage for** | **non-DMP** | | **DMP** | |
| --- | --- | --- | --- | --- |
| **Ace-inhibitors** | N=3870 | 58.92% [35.08%;64.19%] | N=3870 | 60.95% [35.69%;66.55%] |
| **β-blockers **** | **N=3870** | **52.64% [49.13%;55.91%]** | **N=3870** | **61.95% [56.74%;65.90%]** |
| **Statins** | N=3870 | 52.13% [32.19%;55.38%] | N=3870 | 54.18% [32.84%;57.44%] |
| **Anti-platelet-agents** | N=3870 | 70.66% [65.67%;76.43%] | N=3870 | 74.20% [70.04%;83.07%] |
| * **p<0.05** ** **p<0.01** *** **p<0.001**  **Confidence intervals and p-values of the cost differences were derived by bootstrapping the original data set with 1,000 replications (26)** | | | | |

**Supplement Table 2: correlation matrix of Schoenfeld residuals with time**

| **Pearson Correlation Coefficients** | | | | | | | | | |
| --- | --- | --- | --- | --- | --- | --- | --- | --- | --- |
| **Prob > \|r\| under H0: Rho=0** | | | | | | | | | |
| **Number of Observations** | | | | | | | | | |
|  | **Days** | **DMP CAD** | **Sex** | **Smoker** | **Obesity** | **Angina pectoris** | **Arterial occlusive disease** | **Dyslipidemia** | **Conges-tive heart failure** |
| **Days** | 100.000 | 0.03116 | -0.00614 | 0.02684 | 0.01613 | -0.02751 | 0.00464 | 0.01873 | -0.01760 |
|  |  | 0.1146 | 0.7560 | 0.1740 | 0.4140 | 0.1636 | 0.8143 | 0.3430 | 0.3727 |
|  | 7740 | 2566 | 2566 | 2566 | 2566 | 2566 | 2566 | 2566 | 2566 |
|  | | | | | | | | | |
|  | **Arterial hypertonia** | **NYHA** | **DMP COPD** | **DMP type 2 diabetes** | **Stent before (binary** | **Length index hospitalization** | **Age** | **HMG compensations** | **GIMD 2010** |
| **Days** | 0.00720 | -0.00365 | -0.01347 | 0.01194 | 0.01721 | **-0.04594** | 0.01551 | **0.05036** | -0.02367 |
|  | 0.7154 | 0.8533 | 0.4952 | 0.5455 | 0.3836 | **0.0199** | 0.4324 | **0.0107** | 0.2306 |
|  | 2566 | 2566 | 2566 | 2566 | 2566 | **2566** | 2566 | **2566** | 2566 |
| **CAD (Coronary Artery Disease), NYHA (New York Heart Association), DMP (Disease Management Program), COPD (Chronic Obstructive Disease), HMG (Hierarchical Morbidity Group), GIMD 2010 (German Index of Multiple Deprivation 2010)** | | | | | | | | | |

**Supplement Table 3: Kolmogorov-Smirnoff supreme Test**

| **Supremum Test for Proportionals Hazards Assumption** | | |
| --- | --- | --- |
| **Variable** | **Maximum Absolute Value** | **Pr > MaxAbsVal** |
| **Sex** | 0.6801 | 0.7540 |
| **Smokers** | 10.631 | 0.2210 |
| **Obesity** | 0.6380 | 0.8150 |
| **Angina pectoris** | 0.8707 | 0.4950 |
| **Arterial occlusive disease** | 10.512 | 0.5710 |
| **Dyslipidemia** | 10.856 | 0.3490 |
| **Congestive heart failure** | 0.9139 | 0.5860 |
| **Arterial hypertonia** | 0.9902 | 0.5680 |
| **Nyha** | 13.139 | 0.0550 |
| **Nyha** | 0.8615 | 0.4160 |
| **Nyha** | 10.117 | 0.3180 |
| **Nyha** | 0.7864 | 0.6660 |
| **DMP CAD** | 0.9386 | 0.3140 |
| **DMP COPD** | 0.7743 | 0.5340 |
| **DMP type 2 diabetes** | 0.5997 | 0.8520 |
| **Stent before (binary)** | 0.9249 | 0.4850 |
| **Length index hospitalization** | **14.937** | **0.0180** |
| **Age** | 0.8856 | 0.5850 |
| **HMG compensations** | **19.258** | **0.0010** |
| **GIMD 2010** | 0.7780 | 0.5430 |
| **All results are based on 1,000 replications**  **NYHA (New York Heart Association), DMP (Disease Management Program), CAD (Coronary Artery Disease), COPD (Chronic Obstructive Disease), HMG (Hierarchical Morbidity Group), GIMD 2010 (German Index of Multiple Deprivation 2010)** | | |

**Supplement Table 4: correlation matrix of Schoenfeld residuals with time**

| **Pearson Correlation Coefficients** | | | | | | | | | | | | |
| --- | --- | --- | --- | --- | --- | --- | --- | --- | --- | --- | --- | --- |
| **Prob > \|r\| under H0: Rho=0** | | | | | | | | | | | | |
| **Number of Observations** | | | | | | | | | | | | |
|  | **Days** | **DMP CAD** | | **Sex** | | **Smoker** | **Obesity** | **Angina pectoris** | | **Arterial occlusive disease** | | **Dyslipid-emia** |
| **days** | 1.00000 | 0.02936 | | -0.00562 | | 0.02803 | 0.01546 | -0.02634 | | 0.00943 | | 0.02037 |
|  |  | 0.1371 | | 0.7761 | | 0.1558 | 0.4337 | 0.1823 | | 0.6329 | | 0.3022 |
|  | 7740 | 2566 | | 2566 | | 2566 | 2566 | 2566 | | 2566 | | 2566 |
|  | | | | | | | | | | | | |
|  | **Conges-tive heart failure** | **Arterial hypertonia** | | **NYHA** | | **DMP COPD** | **DMP type 2 diabetes** | **Stent before (binary** | | **Length index hospitalization** | | **Age** |
| **days** | -0.01431 | 0.01064 | | 0.00092 | | -0.01573 | 0.01232 | 0.02020 | | **-0.04283** | | 0.01767 |
|  | 0.4686 | 0.5900 | | 0.9627 | | 0.4258 | 0.5327 | 0.3063 | | **0.0301** | | 0.3709 |
|  | 2566 | 2566 | | 2566 | | 2566 | 2566 | 2566 | | **2566** | | 2566 |
|  | | | | | | | | | | | | |
|  | **HMG compensations** | | **GIMD 2010** | | **PDC Ace-inhibitors** | | **PDC β-blockers** | | **PDC Statins** | | **PDC Anti-platelet-agents** | |
| **days** | **0.05858** | | -0.02304 | | **-0.11533** | | **-0.08287** | | -0.03101 | | -0.03269 | |
|  | **0.0030** | | 0.2434 | | **<.0001** | | **<.0001** | | 0.1163 | | 0.0978 | |
|  | **2566** | | 2566 | | **2566** | | **2566** | | 2566 | | 2566 | |
| **CAD (Coronary Artery Disease), NYHA (New York Heart Association), DMP (Disease Management Program), COPD (Chronic Obstructive Disease), HMG (Hierarchical Morbidity Group), GIMD 2010 (German Index of Multiple Deprivation 2010), PDC (proportion of days covered)** | | | | | | | | | | | | |

**Supplement Table 5: Kolmogorov-Smirnoff supreme Test**

| **Supremum Test for Proportionals Hazards Assumption** | | |
| --- | --- | --- |
| **Variable** | **Maximum Absolute Value** | **Pr > MaxAbsVal** |
| **Sex** | 0.6704 | 0.7680 |
| **Smokers** | 10.980 | 0.1920 |
| **Obesity** | 0.6253 | 0.8330 |
| **Angina pectoris** | 0.8443 | 0.5360 |
| **Arterial occlusive disease** | 12.269 | 0.3780 |
| **Dyslipidemia** | 11.431 | 0.3010 |
| **Congestive heart failure** | 0.8293 | 0.7040 |
| **Arterial hypertonia** | 0.9936 | 0.5720 |
| **Nyha** | 13.290 | 0.0530 |
| **Nyha** | 0.8735 | 0.3950 |
| **Nyha** | 0.9951 | 0.3400 |
| **Nyha** | 0.8719 | 0.5330 |
| **DMP CAD** | 0.9148 | 0.3500 |
| **DMPCOPD** | 0.8126 | 0.4810 |
| **DMP type 2 diabetes** | 0.6106 | 0.8360 |
| **Stent before (binary)** | 10.084 | 0.3960 |
| **Length index hospitalization*** | **14.254** | **0.0280** |
| **Age** | 0.9714 | 0.5080 |
| **HMG compensations** | **21.269** | **<.0001** |
| **GIMD 2010** | 0.7814 | 0.5490 |
| **PDC Ace-inhibitors** | **33.590** | **<.0001** |
| **PDC β-blockers** | **27.444** | **<.0001** |
| **PDC Statins** | 14.790 | 0.1050 |
| **PDC Anti-platelet-agents** | **16.403** | **0.0280** |
| **All results are based on 1,000 replications**  **NYHA (New York Heart Association), DMP (Disease Management Program), CAD (Coronary Artery Disease), COPD (Chronic Obstructive Disease), HMG (Hierarchical Morbidity Group), GIMD 2010 (German Index of Multiple Deprivation 2010), PDC (proportion of days covered)** | | |

**Supplement Table 6: Basic Hazard Ratio Modell with time dependent variables**

| **Parameters** |  | **DF** | **Estimate** | **StdErr** | **ChiSq** | **ProbChiSq** | **HazardRatio (CI 95%)** |
| --- | --- | --- | --- | --- | --- | --- | --- |
| **Sex** |  | **1** | **-0.18404** | **0.04261** | **18.6526** | **<.0001** | **0.832 (0.765-0.904)** |
| **Smokers** |  | **1** | **0.53372** | **0.08532** | **39.1303** | **<.0001** | **1.705 (1.143-2.016)** |
| **Obesity** |  | **1** | -0.02899 | 0.05499 | 0.2779 | 0.5981 | 0.971 (0.872-1.082) |
| **Angina pectoris** |  | **1** | **-0.20892** | **0.07366** | **8.0439** | **0.0046** | **0.811 (0.702-0.937)** |
| **Arterial occlusive disease** |  | **1** | **0.19027** | **0.05909** | **10.3677** | **0.0013** | **1.201 (1.077-1.358)** |
| **Dyslipidemia** |  | **1** | -0.03423 | 0.05851 | 0.3423 | 0.5585 | 0.966 (0.862-1.084) |
| **Congestive heart failure** |  | **1** | **0.34743** | **0.05480** | **40.1940** | **<.0001** | **1.415 (1.271-1.576)** |
| **Arterial hypertonia** |  | **1** | **0.11446** | **0.05177** | **4.8893** | **0.0270** | **1.121 (1.013-1.241)** |
| **Nyha** | **1** | **1** | 0.03254 | 0.16944 | 0.0369 | 0.8477 | 1.033 (0.741-1.440) |
| **Nyha** | **2** | **1** | -0.01396 | 0.09536 | 0.0214 | 0.8836 | 0.986 (0.818-1.189) |
| **Nyha** | **3** | **1** | **0.36490** | **0.05864** | **38.7245** | **<.0001** | **1.440 (1.284-1.616)** |
| **Nyha** | **4** | **1** | **0.56550** | **0.04960** | **129.9677** | **<.0001** | **1.760 (1.597-1.940)** |
| **DMP CAD** |  | **1** | **-0.27969** | **0.04005** | **48.7591** | **<.0001** | **0.756 (0.699-0.818)** |
| **DMPCOPD** |  | **1** | **0.27287** | **0.08046** | **11.5004** | **0.0007** | **1.314 (1.122-1.538** |
| **DMP type 2 diabetes** |  | **1** | **0.11407** | **0.04078** | **7.8251** | **0.0052** | **1.121 (1.035-1.214)** |
| **Stent before (binary)** |  | **1** | 0.08282 | 0.05251 | 2.4873 | 0.1148 | 1.086 (0.980-1.214) |
| **Length index hospitalization** |  | **1** | **0.02529** | **0.00319** | **63.0262** | **<.0001** | **1.026 (1.019-1.032)** |
| **Age** |  | **1** | **0.06231** | **0.00259** | **578.7140** | **<.0001** | **1.064 (1.059-1.070)** |
| **HMG compensations** |  | **1** | **0.00834** | **0.00106** | **62.1311** | **<.0001** | **1.008 (1.006-1.010)** |
| **GIMD 2010** |  | **1** | 0.00049 | 0.00261 | 0.0350 | 0.8516 | 1.000 (0.995-1.006) |
| **Days*HMG compensations** |  | **1** | **0.00001** | **0.00000** | **7.4091** | **0.0065** | **1.000 (1.000-1.00)** |
| **Days*Length index hospitalization** |  | **1** | **-0.00002** | **0.00001** | **5.6913** | **0.0170** | **1.000 (1.000-1.000)** |
| **NYHA (New York Heart Association), DMP (Disease Management Program), CAD (Coronary Artery Disease), COPD (Chronic Obstructive Disease), HMG (Hierarchical Morbidity Group), GIMD 2010 (German Index of Multiple Deprivation 2010)** | | | | | | | |

**Supplement Table 7: Proportional Hazard Model with guideline recommended medication and time dependent variables**

| **Parameters** |  | **DF** | **Estimate** | **StdErr** | **ChiSq** | **ProbChiSq** | **HazardRatio (CI 95%)** |
| --- | --- | --- | --- | --- | --- | --- | --- |
| **Sex** |  | **1** | **-0.17664** | **0.04275** | **17.0724** | **<.0001** | **0.838 (0.771-0.911)** |
| **Smokers** |  | **1** | **0.52541** | **0.08550** | **37.7598** | **<.0001** | **1.691 (1.430-2.000)** |
| **Obesity** |  | **1** | -0.05326 | 0.05500 | 0.9376 | 0.3329 | 0.948 (0.851-1.056) |
| **Angina pectoris** |  | **1** | **-0.21153** | **0.07379** | **8.2186** | **0.0041** | **0.809 (0.700-0.935)** |
| **Arterial occlusive disease** |  | **1** | **0.17177** | **0.05936** | **8.3744** | **0.0038** | **1.187 (1.057-1.334)** |
| **Dyslipidemia** |  | **1** | -0.01533 | 0.05888 | 0.0678 | 0.7945 | 0.985 (0.877-1.105) |
| **Congestive heart failure** |  | **1** | **0.34032** | **0.05493** | **38.3787** | **<.0001** | **1.405 (1.262-1.565)** |
| **Arterial hypertonia*** |  | **1** | **0.10460** | **0.05197** | **4.0500** | **0.0442** | **1.110 (1.003-1.229)** |
| **Nyha** | **1** | **1** | 0.06151 | 0.16940 | 0.1319 | 0.7165 | 1.063 (0.763-1.482) |
| **Nyha** | **2** | **1** | -0.00345 | 0.09541 | 0.0013 | 0.9711 | 0.997 (0.827-1.201) |
| **Nyha** | **3** | **1** | **0.36454** | **0.05868** | **38.5944** | **<.0001** | **1.440 (1.283-1.615)** |
| **Nyha** | **4** | **1** | **0.54846** | **0.04965** | **122.0185** | **<.0001** | **1.731 (1.570-1.97)** |
| **DMP CAD** |  | **1** | **-0.27170** | **0.04024** | **45.5970** | **<.0001** | **0.762 (0.704-0.825)** |
| **DMPCOPD** |  | **1** | **0.27639** | **0.08070** | **11.7302** | **0.0006** | **1.318 (1.125-1.544)** |
| **DMP type 2 diabetes** |  | **1** | **0.09551** | **0.04094** | **5.4440** | **0.0196** | **1.100 (1.015-1.192)** |
| **Stent before (binary)*** |  | **1** | **0.10823** | **0.05296** | **4.1765** | **0.0410** | **1.114 (1.004-1.236)** |
| **Length index hospitalization** |  | **1** | **0.02531** | **0.00319** | **62.8490** | **<.0001** | **1.026 (1.019-1.032)** |
| **Age** |  | **1** | **0.06230** | **0.00267** | **542.5946** | **<.0001** | **1.064 (1.059-1.070)** |
| **HMG compensations** |  | **1** | **0.00821** | **0.00108** | **58.3360** | **<.0001** | **1.008 (1.006-1.010)** |
| **GIMD 2010** |  | **1** | -0.00089 | 0.00263 | 0.1145 | 0.7351 | 0.999 (0.994-1.004) |
| **PDC ACE-inhibitors** |  | **1** | **0.00683** | **0.00093** | **53.3784** | **<.0001** | **1.007 (1.005-1.009)** |
| **PDC β-blockers** |  | **1** | **0.00377** | **0.00102** | **13.6218** | **0.0002** | **1.004 (1.002-1.006)** |
| **PDC Statins** |  | **1** | **-0.00200** | **0.00062** | **10.4924** | **0.0012** | **0.998 (0.997-0.999)** |
| **PDC Anti-platelet-agents** |  | **1** | **0.00635** | **0.00089** | **51.5075** | **<.0001** | **1.006 (1.005-1.008)** |
| **Days*HMG compensations*** |  | **1** | **0.00001** | **0.00000** | **5.4648** | **0.0194** | **1.000 (1.000-1.000)** |
| **Days*Length index hospitalization** |  | **1** | **-0.00002** | **0.00001** | **27.2596** | **<.0001** | **1.000 (1.000-1.000)** |
| **Days*PDC ACE-inhibitors** |  |  | **-0.00001** | **0.00000** | **27.2596** | **<.0001** | **1.000 (1.000-1.000)** |
| **Days* β-blockers** |  |  | **-0.00001** | **0.00000** | **11.8095** | **0.0006** | **1.000 (1.000-1.000)** |
| **Days*Anti-platelet agents** |  |  | **-0.00000** | **0.00000** | **1.8456** | **0.1743** | **1.000 (1.000-1.000)** |
| **NYHA (New York Heart Association), DMP (Disease Management Program), CAD (Coronary Artery Disease), COPD (Chronic Obstructive Disease), HMG (Hierarchical Morbidity Group), GIMD 2010 (German Index of Multiple Deprivation 2010), PDC (proportion of days covered)** | | | | | | | |

**Supplement Table 8: Sensitivity analysis with Charlson index as comorbidity measure**

| **Parameters** |  | **DF** | **Estimate** | **StdErr** | **ChiSq** | **ProbChiSq** | **HazardRatio**  **(CI 95%)** |
| --- | --- | --- | --- | --- | --- | --- | --- |
| **Sex** |  | **1** | -0.118143 | 0.042633 | 7.679254 | 0.0056 | 0.889 (0.817-0.966 |
| **Smokers** |  | **1** | **0.459344** | **0.085217** | **29.055080** | **<.0001** | **1.583 (1.340-1.871)** |
| **Obesity** |  | **1** | -0.089097 | 0.054941 | 2.629821 | 0.1049 | 0.915 (0.821-1.019) |
| **Angina pectoris** |  | **1** | **-0.175817** | **0.073486** | **5.724161** | **0.0167** | **0.839 (0.726-0.969)** |
| **Arterial occlusive disease** |  | **1** | **0.133873** | **0.058933** | **5.160207** | **0.0231** | **1.143 (1.019-1.283)** |
| **Dyslipidemia** |  | **1** | -0.092135 | 0.057626 | 2.556300 | 0.1099 | 0.912 (0.815-1.021) |
| **Congestive heart failure** |  | **1** | **0.276294** | **0.054388** | **25.806722** | **<.0001** | **1.318 (1.185-1.467)** |
| **Arterial hypertonia** |  | **1** | 0.025892 | 0.051659 | 0.251207 | 0.6162 | 1.026 (0.927-1.136) |
| **Nyha** | **1** | **1** | -0.021448 | 0.169450 | 0.016022 | 0.8993 | 0.979 (0.702-1.364 |
| **Nyha** | **2** | **1** | -0.099330 | 0.095486 | 1.082132 | 0.2982 | 0.905 (0.751-1.092) |
| **Nyha** | **3** | **1** | **0.246269** | **0.058820** | **17.529486** | **<.0001** | **1.279 (1.140-1.436)** |
| **Nyha** | **4** | **1** | **0.443613** | **0.049659** | **79.802601** | **<.0001** | **1.558 (1.414-1.718)** |
| **DMP CAD** |  | **1** | -0.258573 | 0.040108 | 41.562587 | **<.0001** | 0.772 (0.714-0.835) |
| **DMPCOPD** |  | **1** | **0.190242** | **0.080611** | **5.569561** | **0.0183** | **1.210 (1.033-1.417)** |
| **DMP type 2 diabetes** |  | **1** | -0.118832 | 0.043746 | 7.378835 | 0.0066 | 0.888 (0.815-0.967) |
| **Stent before (binary)** |  | **1** | **0.115832** | **0.051784** | **5.003403** | **0.0253** | **1.123 (1.014-1.243)** |
| **Length index hospitalization** |  | **1** | **0.015900** | **0.002102** | **57.222502** | **<.0001** | **1.016 (1.012-1.020)** |
| **Age** |  | **1** | **0.055418** | **0.002614** | **449.472323** | **<.0001** | **1.057 (1.052-1.062)** |
| **Charlson index** |  | **1** | **0.137157** | **0.007495** | **334.896992** | **<.0001** | **1.147 (1.130-1.164)** |
| **GIMD 2010** |  | **1** | -0.000815 | 0.002629 | 0.096029 | 0.7566 | 0.999 (0.994-1.004) |
| **NYHA (New York Heart Association), DMP (Disease Management Program), CAD (Coronary Artery Disease), COPD (Chronic Obstructive Disease), HMG (Hierarchical Morbidity Group), GIMD 2010 (German Index of Multiple Deprivation 2010)** | | | | | | | |
